# Metabolomics Reveals the Regulatory Mechanism of Antibacterial Fiber Membrane Packaging on the Postharvest Quality of Wax Apple (*Syzygium samarangense*)

**DOI:** 10.3390/foods14213794

**Published:** 2025-11-05

**Authors:** Jiale Zhao, Guanglong Yao, Dongfen Huang, Yue Sun, Jian Chen, Hengfu Huan

**Affiliations:** 1 Key Laboratory of Crop Gene Resources and Germplasm Enhancement in Southern China of Ministry of Agriculture and Rural Affairs & Key Laboratory of Tropical Crops Germplasm Resources Genetic Improvement and Innovation of Hainan Province, Tropical Crops Genetic Resources Institute (TCGRI), Chinese Academy of Tropical Agricultural Sciences (CATAS), Haikou 571101, China; 13461308231@163.com (J.Z.); dongfen.huang@163.com (D.H.); sunyue327724@163.com (Y.S.); 2 College of Food Science and Engineering, Hainan University, Haikou 570228, China; yaoguanglong@126.com

**Keywords:** active packaging, antibacterial mechanism, wax apple, non-targeted metabolomics

## Abstract

Wax apple (*Syzygium samarangense*) is highly perishable postharvest. Even under refrigerated storage conditions, its shelf life typically lasts only about one week. This study developed a novel antibacterial food packaging membrane to extend its shelf life and explored the underlying preservation mechanisms. A composite fiber membrane was fabricated via solution blow spinning (SBS) using polyethylene oxide (PEO) and oxidized sesbania gum (OSG) incorporated with ε-polylysine (ε-PL). The composite membrane demonstrated exceptional antibacterial activity against both *E. coli* and *S. aureus* by disrupting cell wall and membrane integrity, as evidenced by increased protein leakage, alkaline phosphatase activity, and electrical conductivity. Morphological observations through scanning electron microscopy confirmed extensive cellular damage and bactericidal effects. During nine days of ambient storage, the PEO/OSG/PL membrane significantly maintained the postharvest quality of wax apples. This was evidenced by a lower decay index (2.22 ± 0.19) and weight loss rate (5.32 ± 0.16%) compared to the control group, alongside better preservation of firmness (4.11 ± 0.08 N) and color stability. The treatment suppressed respiratory rate and delayed the degradation of soluble solids and titratable acidity. Furthermore, it enhanced antioxidant capacity through higher peroxidase activity and reduced malondialdehyde accumulation, indicating attenuated oxidative stress. Non-targeted metabolomics analysis revealed that the membrane treatment modulated critical metabolic pathways, particularly phenylalanine metabolism and linoleic acid metabolism. These metabolic adjustments contributed to enhanced defense responses and delayed senescence. The results show that the PEO/OSG/ε-PL fiber membrane acts as an effective active packaging material by inhibiting microbial growth and regulating metabolism. This provides a potential method to extend the shelf life of perishable fruits.

## 1. Introduction

Wax apple (*Syzygium samarangense*), also known as foreign pawpaw, water pawpaw, and generation of fog, belongs to the myrtle family Pawpaw and is a non-climacteric tropical fruit grown mainly in China, Thailand, Indonesia, and Malaysia [1]. The wax apple fruit has an attractive appearance; its color is bright, and the flesh is cotton-like, rich in water and trace elements such as calcium, magnesium, protein, sugar, vitamin C, and other natural nutrients. However, wax apples are highly susceptible to spoilage after harvest. This susceptibility is primarily due to two factors. On one hand, the fruit exhibits vigorous physiological metabolism post-harvest. Intense respiration rapidly depletes its stored nutrients and accelerates the aging process. On the other hand, its thin, tender skin is prone to mechanical damage. The resulting micro-wounds provide convenient entry points for microbial infection, further exacerbating the spoilage and deterioration of wax apples [1,2]. Specifically, the microorganisms driving this postharvest decay primarily fall into two categories: decay-causing bacteria and fungal and bacteria. Key fungal pathogens, comprise *Fusarium* sp., *Lasiodiplodia rubropurpurea*, and *Pestalotiopsis microspore*, which are the main causes of wax apple rot [3,4,5]. Meanwhile, bacterial strains associated with post-harvest contamination of wax apples include *Klebsiella pneumoniae* and *Pantoea agglomerans*, these pathogens invade through micro-wounds, decompose pulp sugars to produce organic acids, and induce off-flavors and soft rot. Occasional contaminants, such as *E. coli* and *S. aureus*, may further exacerbate decay risks via cross-contamination [6]. Therefore, making decay control particularly critical.

Low-temperature storage is the most common method for preserving postharvest wax apples. Storage at 12–14 °C with 90–95% relative humidity can extend shelf life by 10–14 days [7]. However, prolonged low-temperature storage can cause irreversible damage to the fruit, such as increased respiratory rate or freezing damage, thereby accelerating fruit aging [8]. In previous studies, chitosan/tannic acid/trivalent iron (CS/TA/Fe^3+^) coatings have shown significant efficacy in maintaining the postharvest quality of wax apples by inhibiting reactive oxygen species (ROS, a type of substance that triggers oxidative stress) accumulation to alleviate oxidative stress [3]. Chen, et al. [9] used exogenous melatonin to preserve wax apples. The results showed that exogenous melatonin effectively delayed the postharvest deterioration of wax apples by regulating the balance between the generation of reactive oxygen species and the antioxidant system. However, more effective methods for preserving wax apples after harvest should be explored and evaluated.

In recent years, antibacterial-agent-incorporated active packaging systems have shown great potential for extending the shelf life of fresh agricultural products [10]. Among them, the fiber membranes produced by solution blow spinning (SBS) technology have the advantages of high encapsulation efficiency, controlled release and excellent material and process compatibility [11]. This kind of fiber membrane can be functionalized with natural antibacterial agents to minimize microbial spoilage without affecting food safety. Sesbania, an important green manure crop, produces sesbania gum (SG), a galactomannan composed of mannose and galactose in a 2:1 ratio [12]. Oxidized field gum (OSG) is derived from natural sesame gum and is inherently non-toxic. It is produced by oxidizing sesbania gum with H_2_O_2_, which can improve the viscosity stability of SG, and enhance its spinnability and stability when blended with spinning aids (such as polyethylene oxide, PEO) and other polymers. PEO is a water-soluble, crystalline polymer characterized by excellent biocompatibility, low toxicity, spinnability, and chemical stability. It has been approved by the FDA as a safe material for food contact applications [13]. Therefore, OSG was blended with the spinning aid PEO to prepare polysaccharide-based fiber membranes. ε-Polylysine (ε-PL) is a natural antimicrobial peptide produced through microbial fermentation that is effective against both Gram-positive and Gram-negative bacteria and is safe for human consumption, exhibiting high safety [14]. In our previous study, a PEO/OSG solution was blow-spun into a fibrous membrane containing ε-PL, which demonstrated good thermal stability and antimicrobial ability, effectively prolonging the storage quality of fresh-cut mango at 4 °C [15]. However, the precise mechanism by which solution blow-spun fiber materials exhibit superior antimicrobial activity, and their effect on the post-harvest quality of wax apples, requires further investigation.

Given the antibacterial properties of this fibrous membrane, we hypothesized that the preservation of wax apples would be improved. Therefore, this study aimed to prepare composite fiber membranes made of PEO, OSG, and ε-PL using SBS technology, evaluate the bacteriostatic activity of these membranes, and investigate the mechanism of bacteriostatic inhibition against food-borne microorganisms. Wax apples were preserved at room temperature (25 ± 1 °C). The weight loss, decay rate, hardness, color, respiration intensity, and total soluble solids content of wax apples were assessed at various times during storage. Moreover, the effects of activated fiber membranes on metabolites and their pathways in wax apple fruit were analyzed using non-targeted metabolomics.

## 2. Materials and Methods

### 2.1. Materials

Polyethylene oxide (PEO, average molecular mass = 2 × 10^6^ Da) was purchased from Shanghai Aladdin Biochemical Technology Co., Ltd. (Shanghai, China). Glycine gum (SG, food grade) was purchased from Xi’an Longmao Biotechnology Co., Ltd. (Xi’an, China). ε-Polylysine (ε-PL) was purchased from Zhengzhou Bainafo Bioengineering Co., Ltd. (Zhengzhou, China). Wax apple (variety ‘Ruby’) was purchased from North and South Fruit Market, Haikou, China. All fruits were acquired on the day of harvest, and specimens of uniform size and coloration, free from any mechanical damage or pest infestation, were selected for the experiment. The bacterial strains *E. coli* and *S. aureus*, were obtained from the Guangdong Microbial Culture Collection Center (Guangzhou, China).

### 2.2. Preparation of a Polyethylene Oxide/Oxidized Tinctorial Gum (PEO/OSG) Composite Fiber Membrane Loaded with ε-PL

Based on previously reported methods [15], composite fiber membranes were prepared using PEO, OSG, and ε-PL as raw materials: the sample without ε-PL was designated as PEO/OSG/PL-0; the sample containing 2 wt% ε-PL was designated as PEO/OSG/PL-2. The composite fiber membrane exhibited an average thickness of 0.07 ± 0.01 mm and a water vapor transmission rate of 1.84 ± 0.03 × 10^−10^ g·s^−1^·m^−1^·Pa^−1^ [15].

### 2.3. Determination of In Vitro Antibacterial Activity and Mechanism of Fiber Membranes

#### 2.3.1. Cell Membrane Integrity

The suspension (10^7^ CFU/mL) was centrifuged at 4000 r/min for 5 min, and the bacterial material was collected and resuspended in sterile water 3 times. Three milliliters of the resuspension was added to the fiber membrane, which was subsequently incubated for 3 h at 37 °C in a constant-temperature incubator. The bacterial suspension was subsequently removed and centrifuged at 8000 r/min for 3 min, and the supernatant was collected to determine the concentration of leakage proteins via the Bradford method [16].

#### 2.3.2. Cell Wall Integrity (AKP)

Alkaline phosphatase (AKP) was measured to monitor cell wall integrity. Add 30 mg of fibrous membrane sample to the prepared bacterial suspension (10^6^ CFU/mL). Centrifuge the bacterial suspension at 4000 rpm for 5 min and collect the cell pellet. Add 0.5 mL of sterile water to the cell pellet, then perform ultrasonic disruption in an ice-water bath to break up the cells. Ultrasound for 3–5 s at a time, repeating four times. Determine alkaline phosphatase (AKP) activity according to the instructions of the AKP Activity Assay Kit (Nanjing Jiancheng, A059-2, Nanjing, China) [17].

#### 2.3.3. Cell Membrane Permeability

Relative conductivity (RC) was measured using a conductivity meter (DDS-307A, Shanghai Yidian Scientific Instruments Co., Ltd., Shanghai, China) using the method of Hao, et al. [18] with appropriate modifications. Two types of bacteria, *E. coli* and *S. aureus*, in the logarithmic growth phase were centrifuged at 5000 rpm and 4 °C for 5 min, the supernatant was discarded, and the suspension was washed with 5% (*v*/*v*) glucose until the conductivity of the suspension was close to the conductivity of 5% glucose, at which point the suspension was considered isotonic. The fiber membrane was added into a 5% (*v*/*v*) glucose solution and mixed, and the conductivity was measured and recorded as L_1_. The fiber membrane was added to the isotonic bacteria and incubated at 37 °C for 6 h. The mixture was removed every 1 h to measure the conductivity, which was recorded as L_2_. The original bacterial mixture suspended in 5% (*v*/*v*) dextrose was boiled for 5 min and cooled, after which the conductivity was measured, which was recorded as L_0_. The relative conductivity was calculated according to the following formula.

(1) $$\mathrm{RC}\;(\%)=\frac{L_{2}-L_{1}}{L_{0}}\;\times \;100\;$$

#### 2.3.4. Observation of Bacterial Micromorphology

The samples were prepared according to the method of Ding, et al. [19] with appropriate modifications. The fiber membrane was added to the bacterial suspension at a concentration of 10^7^ CFU/mL and incubated in a constant-temperature incubator at a speed of 150 rpm and a temperature of 37 °C for 12 h. The bacterial suspension was centrifuged at 3000 r/min for 10 min, washed with sterile water and centrifuged twice. All bacterial cells were fixed with 2.5% (*w*/*v*) glutaraldehyde for 12 h at 4 °C, washed three times by centrifugation with sterile water, and dehydrated in 50, 70, 90, 100, and 100% ethanol sequentially for 15 min. The samples were freeze-dried, and then observed by scanning electron mi croscopy (SEM, JCM-6000plus Neo Scope, JEOL Ltd., Akishima, Tokyo, Japan).

### 2.4. Application of a Composite Fiber Membrane in Wax Apple Preservation

Wax apples of uniform size (60–80 g per fruit), deep red skin, no mechanical damage, and at commercial maturity (vibrant color, wide base opening, thick flesh, minimal spongy texture) were chosen and randomly assigned into four groups, with three replicates in each group. The harvesting standards followed the business standard for wax apples (SB/T 10886-2012) [20]. The samples were categorized into four groups: a control group with no covering (CK group), a group covered with PE (PE group), a group covered with PEO/OSG/PL-0 fiber membrane (PL-0 group), and a group covered with PEO/OSG/PL-2 fiber membrane (PL-2 group). The wax apples were weighed and placed into transparent plastic cups, which were then covered with various films. The wax apples were stored at a temperature of 25 ± 1 °C for a period of 9 days. During this time, they were continuously photographed to monitor and assess the quality indices.

#### 2.4.1. Decay Index, Weight Loss

The decay index of wax apples was evaluated by referring to the method of Guo, Zhang, Goksen, Khan, Ahmad, Zhang and Deng [3].

The weight of each group was measured every two days via the weighing method, and the results were averaged. The rate of weight loss was calculated according to the following formula:

(2) $$\mathrm{Weight}\;\mathrm{loss}\;\mathrm{rate}\;\left(\%\right)\;=\;\frac{m_{1}\;-\;m_{2}}{m_{1}}\;\times \;100$$

where m_1_ is the initial mass (g) of the wax apple fruit and m_2_ is the mass (g) of the wax apple fruit after storage for a certain number of days.

#### 2.4.2. Firmness, Color

Fruit hardness was measured via a texture analyser (TA. XT Plus, Stable Micro Systems Co., Ltd., Godalming, Surrey, UK) with a P/4 cylindrical probe according to the method of Wang, et al. [21]. The pretest speed was set at 0.005 m/s, the test speed at 0.001 m/s, and the penetration depth at 0.01 m. The firmness was measured at three locations on each wax apple fruit: the top, middle, and bottom. Three wax apple fruits were chosen for measurement, and the results were averaged and expressed in newtons (N).

The color of wax apple peel was measured using a colorimeter (WF28, Weifu Optoelectronic Technology Co., Ltd., Shenzhen, China) with the CIE L*a*b* color system, D65 light source, and an 8° viewing angle. For each group, three fruits were randomly chosen, and four points on each fruit were measured. The average value of these measurements was calculated. The measurement points were symmetrically positioned around the equator of the fruits.

#### 2.4.3. Determination of Respiratory Strength

The respiratory strength of the wax apple was determined via a gas analyser (GXH-3010E, Beijing Huayun Analytical Instrument Research Institute Co., Ltd., Beijing, China) according to the methods of Li, et al. [22]. The wax apple was weighed and placed in a clean drying dish, and a probe was inserted into the air at the top of the drying dish. A gas analyser was used to measure the value of the CO_2_ volume fraction in the chamber before and after the vessel was sealed for 2 h. The respiratory strength was calculated as follows:

(3) $$X\;=\;\frac{\left(w_{1}\;-\;w_{0}\right)\;\times \;V\;\times \;19.6}{m\;\times \;t}$$

where X is the respiration intensity (mg CO_2_·kg^−1^·h^−1^); w_0_ is the CO_2_ volume fraction (%) in the cavity before sealing; w_1_ is the CO_2_ volume fraction (%) after sealing; V is the volume of the space in the drying dish (mL); m is the weight of the fruit (g); and t is the interval of measurement (h).

#### 2.4.4. Determination of Total Soluble Solids Content and Titratable Acid (TA) Content

The total soluble solids content in the wax apple homogenate was determined using a digital handheld refractometer (PAL-106, Aoyi Instrument Co., Ltd., Shanghai, China). Each group’s experiment was conducted 10 times.

The acid‒base titration method proposed by Wang, et al. [23] was used: 2.0 g of fruit pulp was weighed and placed in a mortar and pestle, 5 mL of distilled water was added, the mixture was ground to a homogenate under an ice bath. The mixture was then centrifuged at 4 °C and 12,000 rpm for 30 min, and the supernatant was collected. After that, the supernatant was transferred to a volumetric flask and fixed with distilled water to 50 mL, 10 mL of the sample mixture was removed, and 2 drops of phenolphthalein were added to each sample solution. Finally, titration was carried out with 0.01 mol/L NaOH, and the dosage was recorded when the solution became slightly red and did not fade for 30 s. This process was repeated three times, and the average value was calculated. The TA was calculated as follows:

(4) $$\mathrm{TA}(\%)\;=\;\frac{C\;\times \;V_{1}\;\times \;K\;\times \;B\;\times \;100}{V_{0}\;\times \;m}$$

where C is the NaOH concentration (0.01 mol/L); V_1_ is the NaOH dosage (mL); V_0_ is the volume of the liquid sample absorbed during titration (mL); B is the total volume of the liquid sample at a constant volume (mL); m is the mass of the sample (g); and K is the acidity conversion factor (K = 0.067).

#### 2.4.5. Malondialdehyde (MDA) Content

In accordance with the methods of Xu, et al. [24], 1 g of fruit pulp was placed in a mortar, and 5 mL of 5% trichloroacetic acid was added. The mixture was ground into a homogenate and then centrifuged at 4000 r/min for 10 min to obtain the extract. One milliliter of the extract was taken and 1 mL of 0.6% thiobarbituric acid was added. The mixture was thoroughly combined, reacted in boiling water for 20 min, and then centrifuged after quickly cooling to obtain the supernatant. The absorbance was measured at wavelengths of 532 nm, 600 nm, and 450 nm. The formula used to calculate the MDA content is as follows:

(5) $$C_{\mathrm{MDA}}\;=\;\frac{[6.45({\mathrm{OD}}_{532}\;-\;{\mathrm{OD}}_{600})\;-\;0.56\;\times \;{\mathrm{OD}}_{450}]\;\times \;V}{V_{0}\;\times \;m}$$

where CMDA is the content of malondialdehyde in the mixture (μmol·kg^−1^); OD_450_, OD_532_ and OD_600_ are the absorbance values of the reaction system at 450 nm, 532 nm and 600 nm, respectively; V is the total volume of the sample extract (mL); V_0_ is the volume of the sample extract taken at the time of determination (mL); and m is the mass of the sample (g).

#### 2.4.6. Measurement of Polyphenol Oxidase (PPO) Activity

PPO activity was determined with reference to the method of Xiao, et al. [25]. Two grams of wax apple fruit pulp were put into a mortar, and 5 mL of 50 mM phosphate buffer (pH = 7.0, containing 1% polyvinylpyridone) was added. The mixture was ground into a homogenate in an ice bath and then centrifuged at 4 °C and 12,000 rpm for 30 min to extract the enzymes. The resulting supernatant was the enzyme extract (peroxidase (POD), which shared this enzyme extract). A reaction mixture was prepared by adding 1.9 mL of phosphate buffer, 1 mL of catechol, and 0.1 mL of enzyme extract, bringing the total volume to 3.0 mL. This mixture was incubated at 30 °C for 3 min, after which the absorbance at 410 nm was measured. One unit of enzyme activity is defined as an increase of 0.01 absorbance units per minute.

#### 2.4.7. Measurement of Peroxidase (POD) Activity

POD activity was determined with reference to the method of Gautam, et al. [26]. To measure the activity of the enzyme POD, 0.2 mL of enzyme extract, 1.5 mL of 4% guaiacol, and 100 μL of 2% hydrogen peroxide were added to a test tube to start the reaction. The mixture was then thoroughly mixed. After incubating at 25 °C for 3 min, the absorbance at 470 nm was measured. One unit of enzyme activity was defined as an increase of 0.01 in absorbance per minute.

#### 2.4.8. Sensory Evaluation

Ten panel members were recruited from the laboratory, comprising five males and five females, with an average age of 25 years. Wax apples were assessed based on appearance, color, aroma, and taste, with 25 points allocated to each criterion for a total score of 100 points [27] (Table 1). The specific evaluation criteria are as follows:

### 2.5. Metabolomics Analysis

Wax apple samples were vacuum freeze-dried and ground to powder. A 30 mg aliquot of sample powder was weighed and added to the internal standard extraction solution. The sample was then centrifuged at 12,000 rpm for 3 min. The resulting supernatant was collected, filtered through a microporous filter membrane, and stored in an injection vial for UPLC-MS/MS analysis. The chromatographic conditions included the use of a Waters ACQUITY UPLC HSS T3 Column (1.8 µm, 2.1 × 100 mm^2^). The mobile phase consisted of ultrapure water (0.1% formic acid) as solvent A and acetonitrile (0.1% formic acid) as solvent B. The column temperature was maintained at 40 °C, with a mobile phase flow rate of 0.40 mL/min and an injection volume of 4 µL.

### 2.6. Statistical Analysis

The data from this experiment represent the average of three replicates. The results were presented as mean ± standard deviation. Univariate analysis of variance was performed using SPSS Statistics 27 software, with Duncan’s multiple range test for post hoc comparisons. The significance level was set at *p* < 0.05. Graphs were generated using Origin 2021 software.

## 3. Results and Discussion

### 3.1. In Vitro Antibacterial Activity and Mechanism of Fiber Membranes

#### 3.1.1. Analysis of Protein Leakage

**Figure 1:**
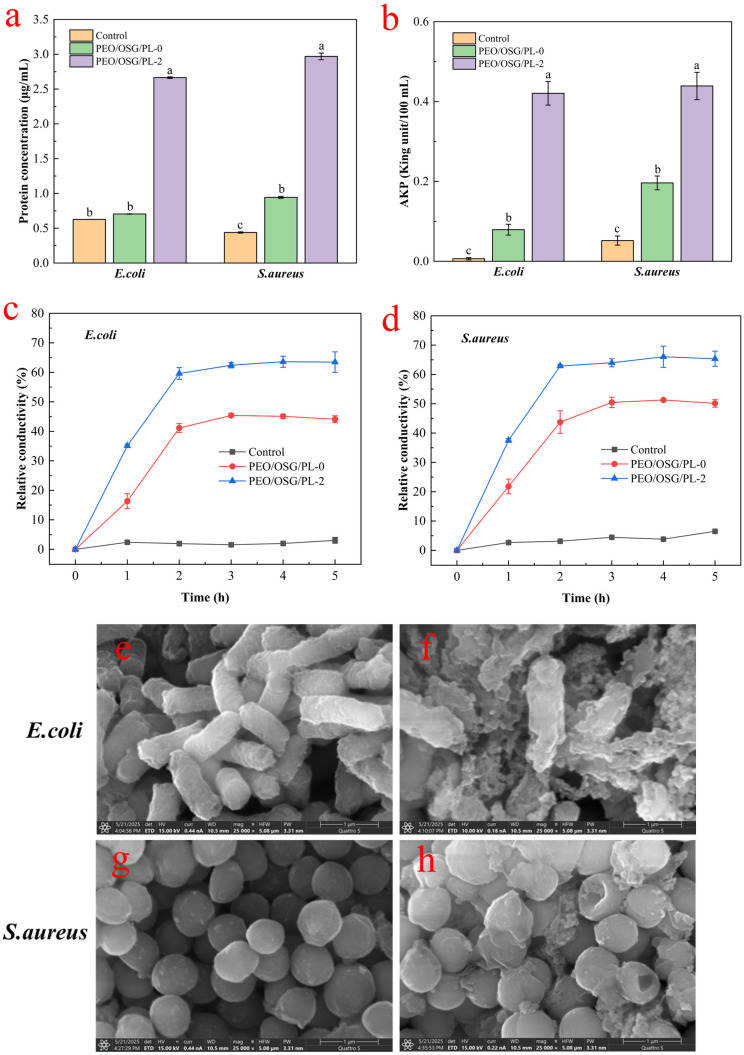
Effect of PEO/OSG/PL fiber membrane on cell membrane integrity (**a**), cell wall integrity (**b**), and cell membrane permeability (**c**,**d**) of *E. coli* and *S. aureus*. (**e**,**g**) SEM images of untreated *E. coli* and *S. aureus*, (**f**,**h**) SEM images of *E. coli* and *S. aureus* treated with PEO/OSG/PL-2 fiber membrane. Vertical bars indicate standard deviation (mean ± SD, *n* = 3). Different letters indicate statistically significant differences (*p* < 0.05).

When a bacterial cell membrane is damaged, macromolecules such as proteins leak from the cell interior [28]. To study the effect of fiber membranes on cell membrane integrity, the amount of protein leaking from the cells was measured, as shown in Figure 1a. The bacterial suspensions without fiber membrane treatment (Control) exhibited the lowest protein content, indicating that the bacterial cell membranes were not affected. The protein contents of *E coli* and *S aureus* in the PEO/OSG/PL-2 fiber membrane treatment group were significantly higher than those in the control group (*p* < 0.05). This may be due to the positive charge of ε-PL in the composite fiber membrane interacting with the negative charge of the phospholipid bilayer in the bacterial cell membrane through electrostatic adsorption, leading to localized deformation of the cell membrane structure [29]. These results indicate that ε-PL in the fiber membrane damages the cell membrane, causing proteins to leak from the membranes of *E. coli* and *S. aureus.*

#### 3.1.2. Analysis of Intracellular AKP Levels

Alkaline phosphatase (AKP) is an enzyme localized in the space between the cell membrane and the cell wall. AKP is not normally secreted into the extracellular environment. However, AKP is released into the extracellular environment when the bacterial cell wall is damaged [30]. Therefore, extracellular AKP activity is commonly used to assess cell wall integrity. As shown in Figure 1b, the control bacterial suspensions of *E. coli* and *S. aureus* exhibited the lowest AKP activity, indicating that the bacterial cell walls were intact. In contrast, the AKP activity in suspensions of both bacterial strains treated with PEO/OSG/PL-2 fiber membranes showed a significant increase (*p* < 0.05). These changes in extracellular AKP activity indicate varying degrees of bacterial cell wall damage. The addition of ε-PL increased the rupture of the bacterial cell membrane structure, resulting in considerable leakage of intracellular AKP. This phenomenon may occur because the cell walls of *E. coli* and *S. aureus* are negatively charged. Meanwhile, ε-PL, being positively charged, can interact with the negatively charged substances in the bacterial cell wall, resulting in surface irregularities on the cell wall [31].

#### 3.1.3. Permeability of the Cell Membrane

Damage to the cell membrane affects its permeability, leading to increased conductivity in the extracellular solution. Changes in the conductivity of bacterial suspensions reflect the effects of antimicrobial substances on bacterial cell membrane permeability [32]. As shown in Figure 1c,d, compared with the control group, the relative conductivity of bacterial suspensions treated with PEO/OSG composite fiber membranes containing ε-PL increased over time and then gradually stabilized. After 5 h of treatment, the relative electrical conductivities of *E. coli* and *S. aureus* in the PL-2 treatment group reached 63.47 ± 3.47% and 65.36 ± 2.60%, respectively, while those in the CK group were only 3.07 ± 1.01% and 6.51 ± 0.41% for *E. coli* and *S. aureus*, respectively. An increase in relative electrical conductivity of the PL-2 cell suspension indicates disruption of the cell membrane, leading to electrolyte leakage and ultimately cell death [18]. Throughout the assay, the group treated with PEO/OSG/PL-2 fiber membrane exhibited higher conductivity than the control group. This suggests that the fiber membrane damaged the bacterial cell membrane, which might inhibit bacterial growth by increasing membrane permeability and disrupting the membrane structure. This effect may be due to the cationic charge carried by ε-PL in the fiber membrane, which adsorbs onto the surface of negatively charged microorganisms and disrupts the cell membrane, resulting in cell death [33].

#### 3.1.4. Microscopic Morphology of Bacteria

The microscopic structure of the two types of bacteria was examined using scanning electron microscopy, as shown in Figure 1e, h. Both *E. coli* and *S. aureus* in the control group displayed smooth and intact cell morphologies. In contrast, cells treated with the fiber membrane exhibited significant morphological changes. *E. coli* cells in the control group were uniformly rod-shaped and structurally intact (Figure 1e). However, *E. coli* treated with PEO/OSG/PL-2 fiber membranes showed roughened surfaces, complete disruption of cellular structure, and abundant debris surrounding the bacteria (Figure 1f). Similarly, *S. aureus* in the control group were spherical with smooth, intact cell morphology (Figure 1g). In contrast, PEO/OSG/PL-2 fiber membrane-treated *S. aureus* exhibited surface damage, depressions, deformations, and the production of irregular material (Figure 1h). These findings indicate that composite fiber membranes incorporating ε-PL (PEO/OSG/PL-2) might suppress the growth of *E. coli* and *S. aureus* by compromising the integrity of their cell membranes, leading to the leakage of intracellular substances.

### 3.2. Experiments on Preserving the Freshness of Wax Apple Seed Pods Via Composite Fiber Membranes

**Figure 2:**
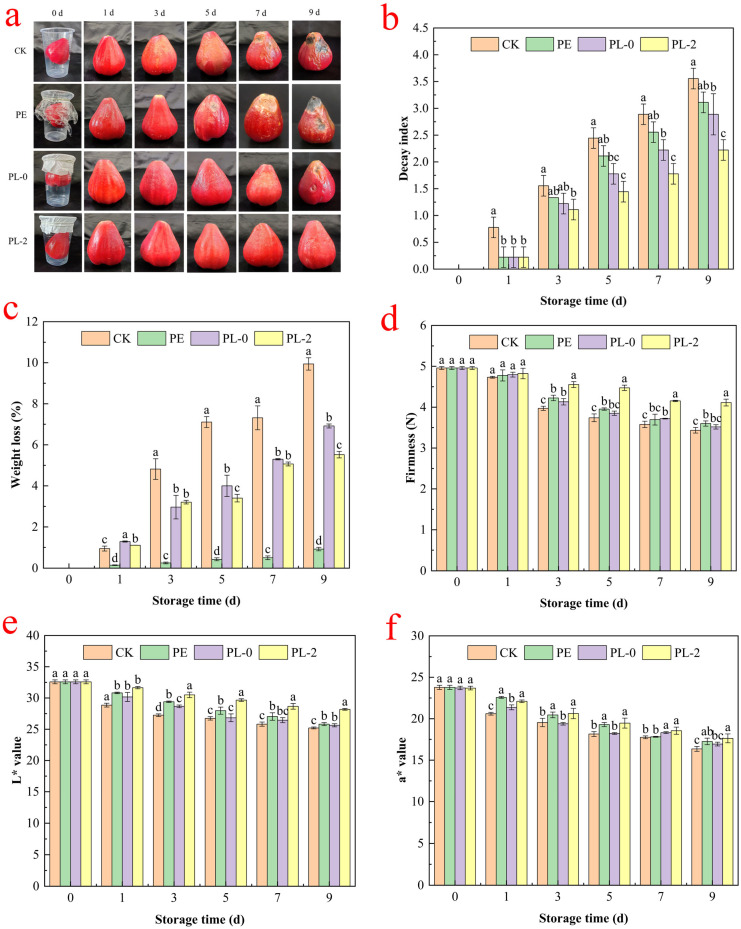
Changes in the appearance (**a**), decay index (**b**), weight loss (**c**), firmness (**d**) and color (**e**,**f**) of wax apple stored for 9 d under different treatments. Vertical bars indicate standard deviation (mean ± SD, *n* = 3). Different letters indicate statistically significant differences (*p* < 0.05).

Figure 2a shows that the CK and PL-0 groups began to exhibit obvious wrinkling and deterioration of wax apple fruit on the third day, with rotting worsening by the ninth day. The PE group started to show signs of decay on the fifth day, while the PL-2 group only exhibited wrinkling and no rotting throughout the entire storage period. This suggests that the PL-2 treatment was the most effective. Therefore, the PEO/OSG/PL-2 fiber membrane effectively prolonged the shelf-life of wax apple fruits.

#### 3.2.1. Decay Index and Weight Loss Rate

Wax apple fruit decay during storage mainly results from factors such as mold infestation and natural aging. Figure 2b shows the changes in decay index of wax apple fruit over the storage period. The decay index increased gradually with storage time, and the decay rate in the CK group was consistently higher than in the other three groups (PE, PL-0, and PL-2) throughout the entire storage period. The PL-2 group consistently had the lowest decay index, significantly lower than the other groups from the seventh day onwards (*p* < 0.05). This effect is attributed to the plastic cups covered with PEO/OSG/PL fiber membranes, which decrease the respiratory intensity of the fruit and release ε-PL at appropriate concentrations, reducing infection by pathogenic bacteria. As shown in Figure 2c, the weight loss rate of all wax apples increased with storage time. The PE group, covered with polyethylene preservation film, exhibited the lowest and slowest increase in weight loss rate. On the 9th day, the weight loss rate was only 0.94 ± 0.12%, primarily due to water vapor produced by respiration and transpiration condensing on the fruit surface. Furthermore, the weight loss rate of wax apples exposed to air (CK group) was significantly higher than that of wax apples covered with fiber membranes (PL-0 and PL-2 groups) (*p* < 0.05). The CK group, being directly exposed to air, experienced higher rates of respiration and transpiration, rapid nutrient consumption, increased water loss, and pericarp wrinkling [34]. The fiber membrane-covered wax apples maintaining a moderate level of water vapor (1.84 ± 0.03 × 10^−10^ g·s^−1^·m^−1^·Pa^−1^), effectively reducing respiration and transpiration. This prevents shriveling and shrinking caused by excessive water loss during storage [23].

#### 3.2.2. Firmness

Fruit softening is one of the primary phenomena of fruit and vegetable ripening and aging, significantly affecting the quality and storage time of produce after harvest. Therefore, the firmness of fruit flesh is a key indicator for assessing the degree of ripening and aging of wax apples [35]. The effects of different treatments on the firmness of wax apples during storage are shown in Figure 2d. Overall, the firmness of wax apples in each group decreased as storage time increased. From day 0 to day 3, the firmness decreased rapidly. From day 3 to day 9, the firmness declined more gradually, continuing until the end of the storage period. The hardness of wax apples in the CK group showed the greatest decrease, falling from 4.96 ± 0.03 N on day 0 to 3.40 ± 0.03 N on day 9. This indicates that exposure to air reduced freshness and accelerated fruit softening. In contrast, after 9 days of storage, the hardness of the PL-2 group remained at 4.07 ± 0.01 N, which was significantly higher than that of the CK, PE, and PL-0 groups. Therefore, the PEO/OSG/PL fiber membrane effectively mitigated the loss of firmness in wax apples. This was mainly due to its ability to inhibit the enzymatic breakdown of cell wall components like pectin and cellulose, thereby reducing fruit softening and preserving firmness [36].

#### 3.2.3. Color

The color of fruit directly affects the appearance of the processed product and consumer acceptance. To evaluate color changes in wax apples during storage, the L* and a* values were recorded, as shown in Figure 2e,f. The L* value indicates peel brightness, and the a* value represents redness. Both values gradually decreased during storage, leading to a loss of luster and color. The L* values of wax apples in the PL-2 group were higher than those of the other three groups (*p* < 0.05). The decrease in brightness is usually caused by the formation of dark tissues and brown spots, which may be related to inappropriate storage conditions and pathogenic bacterial infection [37]. On day 9, the a* values for the CK, PE, PL-0, and PL-2 groups were 16.36 ± 0.29, 17.26 ± 0.40, 16.97 ± 0.32, and 17.51 ± 0.69, respectively. The a* value of PL-2 group was significantly higher than that of CK and PL-0 groups during the late storage period (*p* < 0.05), and slightly higher than that of PE group (*p* > 0.05). Owing to the bacterial inhibitory effect of ε-PL, the PEO/OSG/PL fiber membrane slowed the senescence of wax apples, resulting in higher L* and a* values and fresher fruit in the PL-2 group.

#### 3.2.4. Respiratory Strength

**Figure 3:**
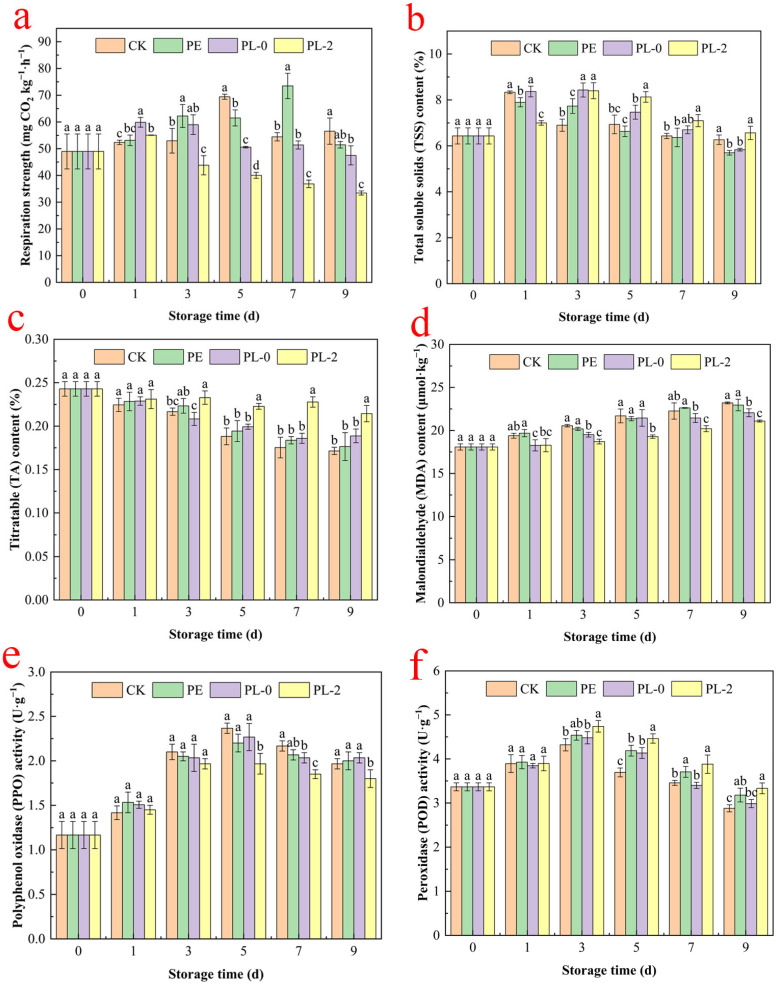
Changes in respiration strength (**a**), total soluble solids (TSS) content (**b**), titratable acid (TA) content (**c**), malondialdehyde (MDA) content (**d**), polyphenol oxidase (PPO) activity (**e**) and peroxidase (POD) activity (**f**) of wax apple stored for 9 d under different treatments. Vertical bars indicate standard deviation (mean ± SD, *n* = 3). Different letters indicate statistically significant differences (*p* < 0.05).

Respiration provides substrates necessary for the physiological metabolism of postharvest fruit and plays a key role in maintaining postharvest quality as well as regulating fruit ripening and senescence [1]. Figure 3a shows the changes in respiration intensity of wax apple fruit during storage. The CK and PE groups exhibited similar respiration trends, both initially increasing and then decreasing, with the PE group consistently having higher respiration intensity than the CK group. This is because PE film, characterized by its dense structure and high water vapor barrier properties, creates an imbalance in the internal packaging environment. It traps transpiration moisture, forming a high-humidity microenvironment, which collectively stimulates the respiration of wax apples, thereby increasing their respiratory intensity [38]. Conversely, the PL-0 and PL-2 groups displayed an overall decreasing trend in respiration intensity, with the PL-0 group maintaining significantly higher respiration than the PL-2 group (*p* < 0.05). This is because the PEO/OSG/PL-2 fiber membrane acts as a barrier to gas exchange, limiting the availability of oxygen for plant tissue respiration. Consequently, it reduces carbon dioxide emissions and slows down metabolic processes [39]. Throughout storage, the CK, PE, and PL-0 groups maintained high respiration intensities, indicating these treatments did not inhibit gas exchange within the wax apples and thus did not suppress respiration. This may explain the rapid proliferation of spoilage microorganisms during the late storage period in these groups.

#### 3.2.5. Total Soluble Solids Content

The total soluble solids content reflects fruit flavor and ripeness and serves as an important indicator of sugar content. As depicted in Figure 3b, total soluble solids content in all treatment groups initially increased and then decreased during storage. The initial increase was due to water loss and further ripening, followed by a gradual decrease caused by respiration and microbial activity, which resulted in the loss of nutrients such as sugars, acids, and vitamins. The decrease in total soluble solids content was less pronounced in the PL-2 group compared to the other groups. This can be attributed to the barrier properties of the fibrous membranes, which restrict oxygen and gas exchange. As a result, the respiration rate of wax apples was reduced, and the consumption of soluble solids was slowed down [40]. Throughout the storage period, the PL-2 group maintained a significantly higher total soluble solids content than the other groups, indicating that the addition of ε-PL to the fiber membrane effectively inhibited microbial growth, which in turn suppressed respiration and metabolic activity in the wax apples, delaying the reduction of total soluble solids.

#### 3.2.6. Titratable Acid Content

Titratable acids are important flavor substances in fruits and serve as one of the main substrates for fruit respiration. A decrease in titratable acid content typically leads to a decline in fruit quality [41]. Figure 3c shows changes in the titratable acid content of wax apple pulp during storage. The titratable acid content in all treatment groups tended to decrease over time, reflecting the consumption of organic acids during respiration, a common occurrence during storage [42]. The impact of various treatments on titratable acidity was inconsistent. The CK group experienced the most significant decline. The PL-2 group slowed the reduction in titratable acid content more effectively than the other groups, with titratable acid levels in the PL-2 group remaining higher than those in the other three groups on day 9. This suggests that the PEO/OSG/PL fiber membrane possesses moderate permeability, which maintains oxygen levels within the microenvironment inside the membrane while reducing the respiration rate of wax apples during storage, thereby slowing titratable acid depletion [43]. Thus, covering wax apples with PEO/OSG/PL fiber membranes can effectively preserve titratable acid content and improve fruit edibility.

#### 3.2.7. MDA Content

MDA is a key product of lipid peroxidation in membranes. Its buildup can harm the cytoplasmic membranes and organelles of fruit, speeding up fruit aging and decay. As can be seen from Figure 3d, during the storage period, the MDA content in the wax apples of all treatment groups steadily increased. After day 3, the MDA content increased rapidly in all groups, indicating elevated membrane lipid oxidation and accelerated fruit senescence. The PL-2 group consistently exhibited significantly lower MDA levels than the other groups throughout storage, indicating that treatment with the PEO/OSG/PL fiber membrane effectively delayed fruit senescence. Additionally, the PL-2 group showed lower MDA content under fiber membrane covering than the other groups at all time points. The PEO/OSG/PL fiber membrane treatment slowed the respiration and metabolism of wax apples, which delayed ripening and reduced tissue damage to some extent [44]. These findings suggest that the PL-2 treatment effectively prevented damage to fruit membrane lipids, thereby delaying senescence.

#### 3.2.8. PPO Activity

PPO is the primary cause of browning in fruits and vegetables, catalyzing the oxidative polymerization of phenolic compounds within fruits to form quinones [45]. As shown in Figure 3e, during storage, the PPO activity of wax apples in all treatment groups initially increased and then decreased. On day 5, the PPO activity of wax apples in all treatment groups reached its maximum value, followed by a decline. Throughout storage, the PL-2 treatment group maintained lower PPO activity than the other three groups. This may be attributed to the PEO/OSG/PL-2 fiber membrane acting as a barrier between polyphenol oxidase and phenolic compounds, thereby minimizing phenolic oxidation. This also highlights the membrane’s ability to preserve cellular membrane integrity [46]. Thus, the PEO/OSG/PL-2 fiber membrane can effectively suppress PPO activity during wax apple storage and delay the peak of enzyme activity.

#### 3.2.9. POD Activity

POD is an important enzyme that detoxifies oxidative free radicals by scavenging ROS. Its activity is closely linked to the processes of fruit maturation and senescence [47]. As shown in Figure 3f, POD activity in wax apples increased initially during storage, reaching peak values on day 3, then declined thereafter. When storage ended, the PL-2 group still had higher POD activity than the other groups, while the CK group had the lowest activity. Specifically, the POD activity in wax apples from the PL-2 group was 1.16 times that of the CK group. The preservation effect of the PEO/OSG/PL-2 fiber membrane treatment on POD activity may be attributed to the maintenance of cell membrane integrity, reduced oxidative damage, the formation of a protective barrier around the fruit surface, and decreased oxygen supply [48]. These factors collectively delay wax apple senescence and reduce ROS production within cells, thus maintaining POD activity in pericarp cells. These results indicate that packaging with PEO/OSG/PL-2 fiber membranes effectively increased antioxidant enzyme activity in wax apples and protected cells from oxidative damage.

#### 3.2.10. Sensory Analysis

**Figure 4:**
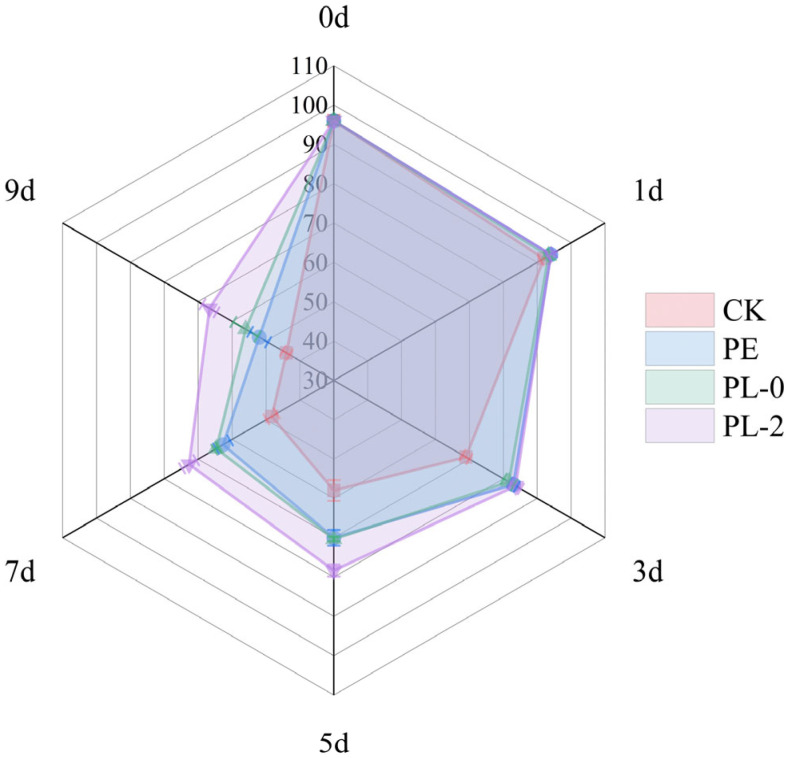
Sensory radar map of wax apple stored for 9 d under different treatments.

The sensory radar chart for wax apples during storage is shown in Figure 4. At the initial storage stages (Day 0 and Day 1), all treatment groups exhibited excellent sensory attributes, including color, aroma, taste, and appearance, with no significant differences, all scoring above 90 points. By the mid-storage period (Day 5), fruits treated with CK exhibited noticeable signs of decay accompanied by browning, leading to significant declines in aroma and taste scores. Their sensory evaluation score was 58 ± 2.65 points. Although the PE and PL-0 groups showed marginally better appearance than the CK group, their preservation effects were limited and failed to effectively inhibit flavor quality deterioration. As storage time extended further, the sensory quality of fruits in the CK and PE groups deteriorated rapidly, with expanded surface rot areas and the development of unpleasant fermentative acidity and putrid odors. Fruits in the PL-0 group also exhibited significant deterioration in color, appearance, and taste. Throughout the entire storage period, the PL-2 group consistently achieved significantly higher sensory scores than all other treatment groups, demonstrating a marked advantage in maintaining post-harvest fruit quality.

### 3.3. Metabolomics Analysis

#### 3.3.1. Multivariate Statistical Analysis

**Figure 5:**
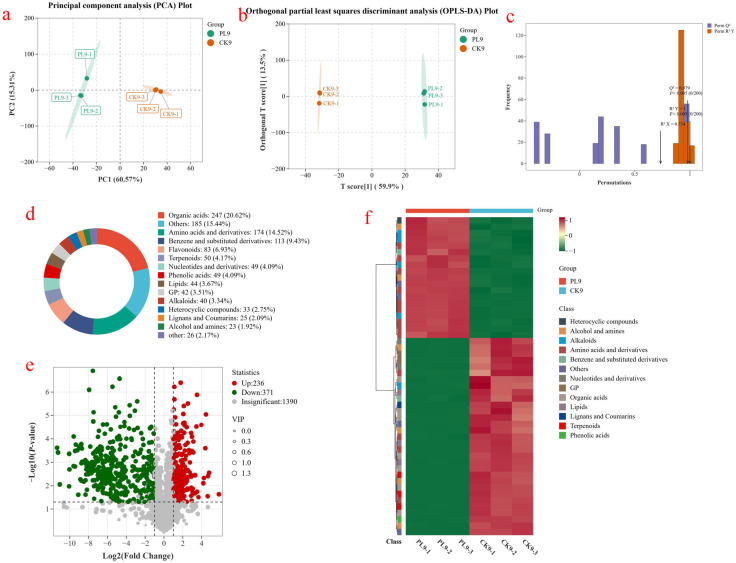
(**a**) Plot of Principal component analysis (PCA) scores of PL9 and CK9 groups; (**b**) Plot of OPLS-DA scores of PL9 and CK9 groups; (**c**) Plot of permutation test of Orthogonal partial least squares discriminant analysis (OPLS-DA) model; (**d**) Species and number of differential metabolites of PL9 and CK9 groups; (**e**) Volcano plots of differential metabolites of PL9 and CK9 groups; (**f**) Clustered heat maps of differential metabolites of PL9 and CK9 groups.

Principal component analysis (PCA) was performed on the metabolites detected in the CK and PL-2 groups of wax apple samples at the 9th day of storage, as shown in Figure 5a. The first principal component accounted for 60.57% of the contributions, while the second principal component accounted for 15.31%, giving a cumulative contribution of 75.88%. This indicates that most of the metabolic information of the wax apple samples was captured, reflecting their main characteristics. The intra-group sample points of the CK9 and PL9 groups were concentrated, and there was no overlap between the inter-group samples, which could be clearly distinguished. This indicates obvious differences in wax apple metabolites packed with the activated fiber membrane.

Orthogonal partial least squares discriminant analysis (OPLS-DA) is a multivariate statistical method for supervised pattern recognition. It decomposes the X-matrix information into components related to Y and irrelevant information, filtering variance variables by removing irrelevant variance, thereby better distinguishing differences between groups. OPLS-DA was performed on wax apple samples from the PL-2 and CK groups on day 9, as shown in Figure 5b. The score plot reveals clear separation between the groups, indicating significant metabolic differences and demonstrating that OPLS-DA effectively distinguishes inter-group differences. Figure 5c presents a validation plot of the OPLS-DA model assessing overfitting. R^2^X and R^2^Y denote the explanatory rates for the X and Y matrices, respectively, while Q^2^ denotes the model’s predictive power. Values closer to 1 indicate higher stability and reliability. For the PL9 and CK9 samples, R^2^X was 0.734, R^2^Y was 1, and Q^2^ was 0.979, all above 0.5. These results indicate that the OPLS-DA model established in this experiment is reliable.

To understand how various metabolites impact the physiological and metabolic processes of wax apples during storage, the metabolites from different treatment groups were examined. The main metabolites differing between the PL9 and CK9 groups included organic acids, other classes, amino acids and their derivatives, benzene and its derivatives, flavonoids, terpenoids, nucleotides and their derivatives, and phenolic acids (Figure 5d). Organic acids were the most abundant, totaling 247 species and accounting for 20.62% of the total metabolites.

Based on the OPLS-DA model, fold change (FC), variable importance in projection (VIP) value, and *p* value were calculated. Metabolites with FC > 2 or FC < 0.5, VIP > 1, and *p* < 0.05 were screened as differential metabolites. The volcano plot of metabolite differences between samples is shown in Figure 5e, revealing 607 significantly different metabolites between the PL9 and CK9 groups. Of these, 236 metabolites were significantly upregulated and 371 were significantly downregulated. Figure 5f shows that the top 50 differential metabolites in abundance in both groups mainly consisted of amino acids and their derivatives, organic acids, benzene and substituted derivatives, lipids, alkaloids, terpenoids, flavonoids, and others.

#### 3.3.2. KEGG Enrichment Analysis of Differential Metabolites

**Figure 6:**
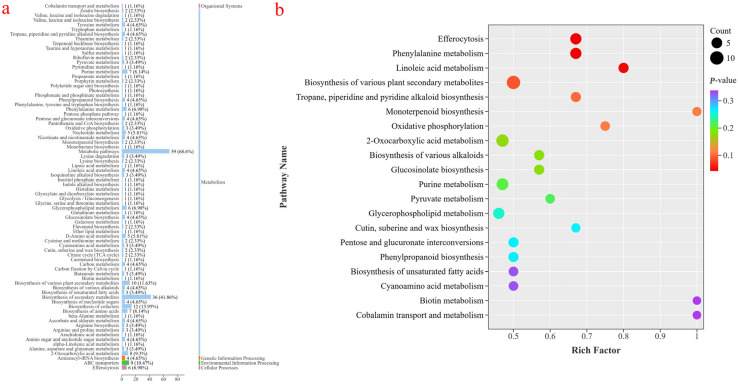
(**a**) Statistical map of KEGG pathways of differential metabolites in PL9 and CK9 groups; (**b**) Bubble map of KEGG enrichment analysis in PL9 and CK9 groups.

As shown in Figure 6a, the 607 different metabolites identified were annotated into 74 metabolic pathways. Among these, 68.6% were categorized under “Metabolic pathways” and 41.86% under “Biosynthesis of secondary metabolites.” KEGG pathway enrichment analysis was further performed to determine differences in metabolic pathways between the CK9 and PL9 groups, with enriched pathways shown in Figure 6b. Comparing PL9 and CK9 samples, differential metabolites were mainly enriched in efferocytosis, phenylalanine metabolism, linoleic acid metabolism, biosynthesis of various plant secondary metabolites, and tropane, piperidine, and pyridine alkaloid biosynthesis, among others. Based on the *p*-value and enrichment degree, two of the most relevant metabolic pathways were identified: phenylalanine metabolism and linoleic acid metabolism.

#### 3.3.3. Analysis of Main Metabolic Pathways

**Figure 7:**
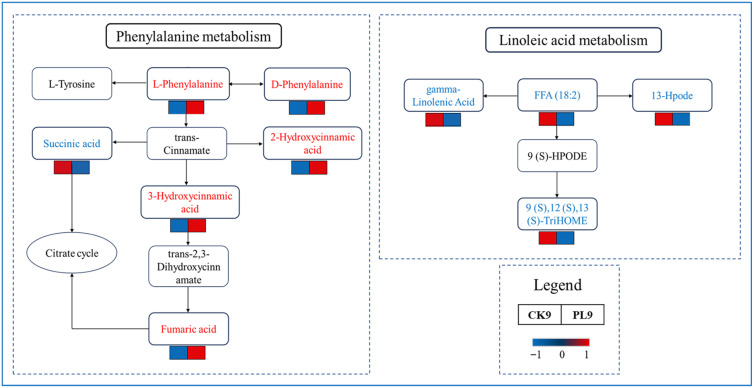
Interrelationship diagram of metabolites related to phenylalanine metabolism and linoleic acid metabolism.

Figure 7 presents the metabolic pathway diagrams for phenylalanine metabolism and linoleic acid metabolism.

Phenylalanine metabolism is a key metabolic pathway in plants, which includes the transformation of several metabolites. The differential metabolites involved in this pathway in this study included succinic acid, 2-hydroxycinnamic acid, 3-hydroxycinnamic acid, D-phenylalanine, L-phenylalanine, and fumaric acid. Among these, the expression level of succinic acid was downregulated in the PL9 group, whereas the expression levels of the other five metabolites were upregulated in the PL9 group. Succinic acid is directly involved in energy metabolism, represented by the citrate cycle; therefore, it can be demonstrated that fiber membrane packaging treatment reduces energy and material losses in wax apple fruits. Succinic acid attenuates the citrate cycle and may lead to the accumulation of fumaric acid due to slowed downstream metabolism. Fumaric acid, as an organic acid, inhibits ROS and contributes to fruit quality [49]. Phenylalanine may serve as a starting material for the production of aromatic compounds in plants, and it triggers the phenylalanine metabolic pathway. It can be transformed into L-tyrosine, which participates in the synthesis of other phenolic compounds. The PL9 group promotes the accumulation of D-phenylalanine and L-phenylalanine, benefiting the secondary anabolic pathways in plants. Additionally, 2-hydroxycinnamic acid and 3-hydroxycinnamic acid were significantly increased in the PL9 group; these belong to the phenolic acid group, and these phenolic compounds possess strong antioxidant properties. Their upregulation enhanced the antioxidant capacity of wax apple fruits, helping protect them from oxidative stress damage.

In addition, the PL9 group activated fiber membrane treatment significantly downregulated several lipid peroxides in the linoleic acid metabolic pathway. Specifically, the levels of 9(S), 12(S), 13(S)-TriHOME, 13-Hpode, gamma-linolenic acid, and FFA (18:2) (linolenic acid) were significantly reduced in the PL9 group. Reduced levels of these metabolites may help protect cell membrane integrity, maintain cellular osmotic pressure, and inhibit cell membrane lipid peroxidation, thereby maintaining the quality of wax apple fruits [50].

## 4. Conclusions

This study investigated the antibacterial mechanisms of PEO/OSG/PL composite fiber membranes prepared by solution blow spinning against *E. coli* and *S. aureus*, as well as their effects on preserving wax apples. The results indicate that these composite fiber membranes disrupt the integrity of bacterial cell walls and membranes, inducing cell lysis, membrane damage, and ultimately bacterial death. In wax apple preservation, this active packaging material effectively delays fruit quality deterioration by primarily inhibiting respiratory metabolism and oxidative stress responses. Non-targeted metabolomics analysis further confirms that the fiber membrane regulates phenylalanine and linoleic acid metabolism, thereby slowing fruit senescence at the metabolic level. This study presents a potential preservation method for wax apples, providing a theoretical foundation for the future development of preservation strategies for wax apples and other perishable fruits.

The raw materials PEO, OSG, and ε-PL used in the preparation of the fiber membranes in this study are not included in the “eight major categories of common food allergens” listed by the Codex Alimentarius Commission (CAC), which include eggs, fish, milk, peanuts, shellfish, soybeans, tree nuts, and wheat [51]. Furthermore, the fiber membranes contain no additional allergenic additives such as colorants or flavorings, resulting in a low risk of allergic reactions. It should be noted that the packaging method employed in this study involves covering the rim of plastic cups with a fiber membrane, which functions as a non-contact barrier. This design ensures that the fiber membrane has virtually no direct contact with the surface of wax apples during storage, significantly reducing the physical possibility of material transfer. Even if accidental contact occurs during subsequent handling, the PEO/OSG/PL-2 fiber membrane’s excellent water solubility allows any residue on the fruit to be removed through routine rinsing with clean water before consumption [15]. Should this material be developed into a commercial product in the future, the packaging should include a warning label stating “Rinse before consumption.” Naturally, in-depth research into whether the fiber membrane components could permeate into the fruit under prolonged contact will be a key focus of our subsequent work. Additionally, quantitative detection of mold and total microbial load on wax apple surfaces during storage will be part of our subsequent work to more comprehensively evaluate the efficacy of the film against primary spoilage microorganisms.

## Figures and Tables

**Table 1:** Sensory evaluation criteria for wax apples.

Parameter	Score Range	Description
Appearance	21–25	Fruit is plump, well-formed, with a smooth surface, showing no wrinkling, depressions, or disease spots.
	16–20	Fruit is generally plump, with slight irregularity in shape. The surface may exhibit very slight wrinkling or a few minor spots.
	11–15	Slightly dehydrated fruit with noticeable wrinkling or deformation, and a few minor blemishes.
	0–10	Severely wrinkled, shriveled, or collapsed fruit with extensive blemishes that significantly impair appearance.
Color	21–25	Bright red skin with vivid, uniform coloration and glossy sheen, free of browning.
	16–20	Relatively bright color, but slightly uneven or slightly dulled, with reduced gloss and very slight browning.
	11–15	Noticeably dulled or lost luster, with distinct brown spots or uneven coloring.
	0–10	Dull color, extensive browning or abnormal yellowish-brown discoloration, completely lacking luster.
Aroma	21–25	Exhibits a rich, fresh aroma characteristic of wax apples, with no off-flavors.
	16–20	Features a distinct wax apple aroma, though relatively faint, or may carry a very slight grassy note.
	11–15	Aroma is weak, or may exhibit noticeable grassy or alcoholic notes.
	0–10	Absence of fruit aroma, accompanied by pronounced rancid, alcoholic, musty, or other unpleasant off-flavors.
Taste	21–25	Crisp texture, abundant juice, balanced sweetness and acidity, pure and rich flavor with no off-flavors.
	16–20	Fairly crisp texture, adequate juice, slightly sour or bland, good flavor.
	11–15	Soft and mushy texture, insufficient juiciness, uneven sweetness and acidity, weak flavor, or slight off-flavor.
	0–10	Soft and mushy or dry and astringent texture, very little juice, excessively sour, bitter, or other strong off-flavors, unfit for normal consumption.

## Data Availability

The original contributions presented in this study are included in the article. Further inquiries can be directed to the corresponding authors.

## References

[1] Zhang Y.-x., Wang Y., Chen F.-h., He F., Wu G.-b., Zhang S., Lin H.-t. (2023). Exogenous nitric oxide inhibits the respiratory metabolism of postharvest wax apple fruit and its role in the delayed cottony softening. Sci. Hortic..

[2] Wei W., Zhang N., Li B., Zhao T., Cheng C., Chen C., Deng H., Yan R. (2024). Simulated logistical transport effects on textural change of wax apple using different packaging types during storage time. Food Packag. Shelf Life.

[3] Guo J., Zhang Z., Goksen G., Khan M.R., Ahmad N., Zhang W., Deng H. (2025). Application of tannic acid and Fe_3+_ crosslinking-enhanced chitosan films for wax apple (*Syzygium samarangense*) preservation. Int. J. Biol. Macromol..

[4] Meng C.-R., Zhang Q., Yang Z.-F., Geng K., Zeng X.-Y., Thilini Chethana K., Wang Y. (2021). *Lasiodiplodia syzygii* sp. nov. (*Botryosphaeriaceae*) causing post-harvest water-soaked brown lesions on *Syzygium samarangense* in Chiang Rai, Thailand. Biodivers. Data J..

[5] Health E.P.o.P., Bragard C., Baptista P., Chatzivassiliou E., Di Serio F., Gonthier P., Jaques Miret J.A., Justesen A.F., MacLeod A., Magnusson C.S. (2023). Pest categorisation of *Pestalotiopsis microspora*. EFSA J..

[6] Esua J.O., Chin N.L., Yusof Y.A., Sukor R. (2017). Antioxidant Bioactive Compounds and Spoilage Microorganisms of Wax Apple (*Syzygium samarangense*) during Room Temperature Storage. Int. J. Fruit Sci..

[7] Khandaker M.M., Boyce A.N. (2016). Growth, distribution and physiochemical properties of wax apple (*Syzygium samarangense*): A Review. Aust. J. Crop Sci..

[8] Lee Y.-C., Chang C.-W., Hsu M.-C., Chung H.-Y., Liang Y.S. (2023). Effects of different concentrations of oxygen used for storage on the postharvest physiology and quality of wax apple (*Syzygium samarangense* Blume Merr. & L. M. perry cv. pink). Sci. Hortic..

[9] Chen Y., Zhang Y., Nawaz G., Zhao C., Li Y., Dong T., Zhu M., Du X., Zhang L., Li Z. (2020). Exogenous Melatonin Attenuates Post-Harvest Decay by Increasing Antioxidant Activity in Wax Apple (*Syzygium samarangense*). Front. Plant Sci..

[10] Shi C., Fang D., Huang C., Zhou A., Lu T., Wang J., Song Y., Lyu L., Wu W., Li W. (2023). Active electrospun nanofiber packaging maintains the preservation quality and antioxidant activity of blackberry. Postharvest Biol. Technol..

[11] Yang Y., Zheng S., Liu Q., Kong B., Wang H. (2020). Fabrication and characterization of cinnamaldehyde loaded polysaccharide composite nanofiber film as potential antimicrobial packaging material. Food Packag. Shelf Life.

[12] Devkar P., Nangare S., Zawar L., Shirsath N., Bafna P., Jain P. (2023). Design of polyacrylamide grafted sesbania gum-mediated pH-responsive IPN-based microbeads for delivery of diclofenac sodium: In-vitro-in-vivo characterizations. Int. J. Biol. Macromol..

[13] Zhu Y., Cui H., Li C., Lin L. (2019). A novel polyethylene oxide/Dendrobium officinale nanofiber: Preparation, characterization and application in pork packaging. Food Packag. Shelf Life.

[14] Yong Y., Gu Y., Ahmad H.N., Wang L., Wang R., Zhu J. (2024). Design and characterization of tannic acid/E-polylysine biocomposite packaging films with excellent antibacterial and antioxidant properties for beef preservation. Food Chem..

[15] Zhao J., Huan H., Yang T., Chen J., Yao G. (2025). Fibrous membranes of poly(ethylene oxide)/Sesbania gum oxide/ε-poly(lysine): An influence on its structure. Food Chem..

[16] Zhang L., Wang Y., Ling S., Yuan M., Sun Q., Dong X. (2025). Antibacterial mechanism of chitooligosaccharides against specific spoilage organisms in chilled processed fish paste products. Food Control.

[17] Yuan Y., Liu L., Guo L., Wang L., Liu Y. (2023). Antibacterial mechanism of rose essential oil against Pseudomonas putida isolated from white *Hypsizygus marmoreus* at cellular and metabolic levels. Ind. Crops Prod..

[18] Hao Y., Zhang M., Wang L., Tao N., Li L., Zhu W., Xu C., Deng S., Wang Y. (2022). Mechanism of antimicrobials immobilized on packaging film inhabiting foodborne pathogens. LWT.

[19] Ding J., Dwibedi V., Huang H., Ge Y., Li Y., Li Q., Sun T. (2023). Preparation and antibacterial mechanism of cinnamaldehyde/tea polyphenol/polylactic acid coaxial nanofiber films with zinc oxide sol to *Shewanella putrefaciens*. Int. J. Biol. Macromol..

[20] (2012). Specification for the Circulation of Wax Apples.

[21] Wang T., Zhai X., Huang X., Li Z., Zhang X., Zou X., Shi J. (2023). Effect of different coating methods on coating quality and mango preservation. Food Packag. Shelf Life.

[22] Li X., Peng S., Yu R., Li P., Zhou C., Qu Y., Li H., Luo H., Yu L. (2022). Co-Application of 1-MCP and Laser Microporous Plastic Bag Packaging Maintains Postharvest Quality and Extends the Shelf-Life of Honey Peach Fruit. Foods.

[23] Wang M., Huang D., Sun Y., Yao G., Huan H., Chen J. (2024). Antibacterial Activity of Modified Sesbania Gum Composite Film and Its Preservation Effect on Wampee Fruit (*Clausena lansium* (Lour.) Skeels). Foods.

[24] Xu C., Zhang X., Liang J., Fu Y., Wang J., Jiang M., Pan L. (2022). Cell wall and reactive oxygen metabolism responses of strawberry fruit during storage to low voltage electrostatic field treatment. Postharvest Biol. Technol..

[25] Xiao H., Zhang S., Xi F., Yang W., Zhou L., Zhang G., Zhu H., Zhang Q. (2023). Preservation effect of plasma-activated water (PAW) treatment on fresh walnut kernels. Innov. Food Sci. Emerg. Technol..

[26] Gautam A., Gill P.P.S., Singh N., Jawandha S.K., Arora R., Singh A. (2024). Composite coating of xanthan gum with sodium nitroprusside alleviates the quality deterioration in strawberry fruit. Food Hydrocoll..

[27] Chang H., Li K., Ye J., Chen J., Zhang J. (2024). Effect of Dual-Modified Tapioca Starch/Chitosan/SiO_2_ Coating Loaded with Clove Essential Oil Nanoemulsion on Postharvest Quality of Green Grapes. Foods.

[28] Cui R., Liu F.-Y., Wu H. (2025). Enhancement of antibacterial activity of cinnamaldehyde against *Bacillus cereus* by nanoemulsion and its application in milk. Food Biosci..

[29] Gao S., Zhang Y., Wang J., Jiang J., Zhai X., Wang W., Hou H. (2022). Influence of starch content on the physicochemical and antimicrobial properties of starch/PBAT/ε-polylysine hydrochloride blown films. Food Packag. Shelf Life.

[30] Wang S., Liu S., Hao G., Zhao L., Lü X., Wang H., Wang L., Zhang J., Ge W. (2022). Antimicrobial activity and mechanism of isothiocyanate from *Moringa oleifera* seeds against *Bacillus cereus* and *Cronobacter sakazakii* and its application in goat milk. Food Control.

[31] Guan G., Zhang L., Zhu J., Wu H., Li W., Sun Q. (2021). Antibacterial properties and mechanism of biopolymer-based films functionalized by CuO/ZnO nanoparticles against *Escherichia coli* and *Staphylococcus aureus*. J. Hazard. Mater..

[32] Qi J., Xu H., Wu X., Lv Z., Yue T., Yang H., Yuan Y. (2025). Antimicrobial activity and mechanism of protocatechualdehyde against *Alicyclobacillus* spp. And its effect on apple juice physicochemical properties. Food Control.

[33] Wang Z., Tian Y., Wang Q., Guo T., Yuan Y., Yue T., Jia H., Ge Q., Zhao Z., Cai R. (2023). Antimicrobial activity and mechanism of preservatives against *Alicyclobacillus acidoterrestris* and its application in apple juice. Int. J. Food Microbiol..

[34] Zuo X., Wang J., Li Y., Zhang J., Wu Z., Jin P., Cao S., Zheng Y. (2025). Recent advances in high relative humidity strategy for preservation of postharvest fruits and vegetables: A comprehensive review. Food Chem..

[35] Yang S., Miao Q., Huang Y., Jian P., Wang X., Tu M. (2020). Preparation of cinnamaldehyde-loaded polyhydroxyalkanoate/chitosan porous microspheres with adjustable controlled-release property and its application in fruit preservation. Food Packag. Shelf Life.

[36] Shen C., Yang X., Wang D., Li J., Zhu C., Wu D., Chen K. (2024). Carboxymethyl chitosan and polycaprolactone-based rapid in-situ packaging for fruit preservation by solution blow spinning. Carbohydr. Polym..

[37] Shao P., Zhang H., Niu B., Jiang L. (2018). Antibacterial activities of R-(+)-Limonene emulsion stabilized by *Ulva fasciata* polysaccharide for fruit preservation. Int. J. Biol. Macromol..

[38] Xiao M., Leung A.Y.K., Zhu Z., Zhu L., Xiao K. (2025). Optimization of the extraction of duckweed protein and its application as an antioxidant composite membrane in blueberry preservation. Int. J. Biol. Macromol..

[39] Chiumarelli M., Hubinger M.D. (2012). Stability, solubility, mechanical and barrier properties of cassava starch—Carnauba wax edible coatings to preserve fresh-cut apples. Food Hydrocoll..

[40] Liang F., Liu C., Geng J., Chen N., Lai W., Mo H., Liu K. (2024). Chitosan–fucoidan encapsulating cinnamaldehyde composite coating films: Preparation, pH-responsive release, antibacterial activity and preservation for litchi. Carbohydr. Polym..

[41] Geng C., Liu X., Ma J., Ban H., Bian H., Huang G. (2023). High strength, controlled release of curcumin-loaded ZIF-8/chitosan/zein film with excellence gas barrier and antibacterial activity for litchi preservation. Carbohydr. Polym..

[42] Yang H., Li L., Li C., Xu Z., Tao Y., Lu J., Xia X., Tan M., Du J., Wang H. (2024). Multifunctional and antimicrobial carboxymethyl cellulose-based active hydrogel film for fruits packaging and preservation. Food Biosci..

[43] Yang Z., Guan C., Zhou C., Pan Q., He Z., Wang C., Liu Y., Song S., Yu L., Qu Y. (2023). Amphiphilic chitosan/carboxymethyl gellan gum composite films enriched with mustard essential oil for mango preservation. Carbohydr. Polym..

[44] Huang J., Wu W., Niu B., Fang X., Chen H., Wang Y., Gao H. (2023). Characterization of *Zizania latifolia* polysaccharide-corn starch composite films and their application in the postharvest preservation of strawberries. Lwt-Food Sci. Technol..

[45] Xin Y., Yang C., Zhang J., Xiong L. (2023). Application of Whey Protein-Based Emulsion Coating Treatment in Fresh-Cut Apple Preservation. Foods.

[46] Ding J., Hao Y., Liu B., Chen Y., Li L. (2023). Development and Application of Poly (Lactic Acid)/Poly (Butylene Adipate-Co-Terephthalate)/Thermoplastic Starch Film Containing Salicylic Acid for Banana Preservation. Foods.

[47] Liu G., Chen B., Liu H., Wang X., Zhang Y., Wang C., Liu C., Zhong Y., Qiao Y. (2023). Effects of Hydroxyethyl Cellulose and Sulfated Rice Bran Polysaccharide Coating on Quality Maintenance of Cherry Tomatoes during Cold Storage. Foods.

[48] Zhao J., Wang Y., Li J., Lei H., Zhen X., Gou D., Liu T. (2023). Preparation of chitosan/Enoki mushroom foot polysaccharide composite cling film and its application in blueberry preservation. Int. J. Biol. Macromol..

[49] Tan Y., Gao M., Li L., Jiang H., Liu Y., Gu T., Zhang J. (2024). Functional components and antioxidant activity were improved in ginger fermented by *Bifidobacterium adolescentis* and *Monascus purpureus*. LWT.

[50] Bu H., Huang X., Huang Q., Cheng P., Dong T., Yun X. (2024). Integrated proteomics and metabolomics analysis reveals the molecular mechanism of PP/PBAT-modified atmosphere packaging for the preservation of *Allium mongolicum* Regel. Postharvest Biol. Technol..

[51] Monaci L., De Angelis E., Montemurro N., Pilolli R. (2018). Comprehensive overview and recent advances in proteomics MS based methods for food allergens analysis. TrAC Trends Anal. Chem..

